# Hemoglobin Level and Associated Factors among Pregnant Women in Rural Southwest Ethiopia

**DOI:** 10.1155/2021/9922370

**Published:** 2021-05-19

**Authors:** Melesse Niguse Kuma, Dessalegn Tamiru, Tefera Belachew

**Affiliations:** ^1^Department of Nutrition and Dietetics, Jimma University, Jimma, Ethiopia; ^2^School of Graduate Studies, Jimma University, Jimma, Ethiopia

## Abstract

**Background:**

Anemia is a situation in which the number and size of red blood cells, or the concentration of hemoglobin, fall below established cut-off values. Low hemoglobin level during pregnancy favors the alteration of placental angiogenesis and resulted in restricting the availability of nutrients to the fetus and consequently causing fetal growth retardation and low weight at birth. This study is aimed at assessing the hemoglobin level and associated factors among pregnant women in rural communities of Jimma zone, Southwest Ethiopia.

**Methods:**

A community-based cross-sectional study design was carried out among 367 pregnant women from June 1 to 30, 2020. Systematic random sampling was used to select study subjects. Hemoglobin level was measured by using HemoCue HB 301. An interviewer-administered structured questionnaire was used to collect the data. Descriptive statistics were used to describe the study subjects. A multivariable linear regression model was employed after the linearity, normality, multicollinearity, and homoscedasticity assumptions were checked. The unstandardized beta (*β*) coefficient along with a 95% confidence interval was computed to estimate the association between explanatory and dependant variables. Statistical significance was declared at *P* value < 0.05.

**Results:**

The mean (± SD) hemoglobin level of the respondents was 12.66 (±1.44) g/dl. The overall magnitude of anemia (hemoglobin level < 11 g/dl) among pregnant women was found to be 85 [23.16%, (95% CI: 18.3%-27.5%)]. Meal frequency [*β* = 0.40, (95% CI: 0.12, 0.69), *P* = 0.005], interpregnancy interval [*β* = 0.08, (95% CI: 0.02, 0.15), *P* = 0.007], mid-upper arm circumference measurement [*β* = 0.13, (95% CI: 0.07, 0.20), *P* ≤ 0.001], own fruits/vegetable [*β* = 0.55, (95% CI: 0.79, 0.31), *P* ≤ 0.001], coffee consumption [*β* = −1.00, (95% CI: -1.31, -0.68), *P* ≤ 0.001], and having history of still birth [*β* = −0.63, (95% CI: -1.06, -0.20), *P* = 0.004**]** were significantly associated with the hemoglobin level of pregnant women.

**Conclusions:**

Anemia was identified to be a moderate public health problem in the study area. Therefore, nutritional counseling should focus on the necessity of at least one extra meal, promotion of fruits/vegetable consumption, and improving the nutritional status of the women during antenatal care follow-up. Moreover, early screening and management of women with a history of stillbirth for anemia are also essential.

## 1. Background

Anemia is a situation in which the number and size of red blood cells, or the hemoglobin concentration, fall below established cut-off values, consequently impairing the capacity of the blood to transport oxygen to the body [1]. The World Health Organization defined anemia during pregnancy as a hemoglobin level of <11 g/dl [2]. Even though measurement of blood hemoglobin concentration alone does not determine the cause of anemia, it is the reliable indicator of anemia at a population level [3]. Globally, about 38% of pregnant women are anemic of which almost two-thirds were from developing countries [3]. Low hemoglobin level or anemia in pregnancy is a public health problem associated with many adverse birth outcomes such as premature delivery, low birth weight, and increased newborn and maternal mortality [4, 5].

Iron deficiency anemia is the most common cause of anemia accounted for more than 50% of all types of anemia in women, resulted from prolonged negative iron balance, caused by poor gut absorption or inadequate dietary iron intake, increased body demand of iron during pregnancy or growth periods, and increased iron loss [3, 6]. Iron is crucial for the fundamental metabolic process in cells and organisms' functions, including respiration, energy production, DNA production, and cell proliferation [7]. During pregnancy, low hemoglobin level favors the alteration of placental angiogenesis and resulted in restricting the availability of oxygen to the fetus and consequently causing fetal growth restriction and low birth weight [8]. Evidence has shown that there is a U-shaped curve relationship between maternal hemoglobin concentration and risk of adverse birth outcomes; however, the association varied by trimester [9]. Studies revealed that both low and high hemoglobin concentrations during pregnancy were associated with the risk of adverse birth outcomes through several mechanisms. Therefore, only an adequate supply of iron during pregnancy is essential [10]. The link between low maternal hemoglobin concentration and adverse birth outcomes is more clear when it is measured during the first trimester of pregnancy, whereas the high hemoglobin concentration is associated during all three trimesters of pregnancy [9]. To reduce this risk, WHO recommends a universal daily supplementation of oral elemental iron 30-60 mg and folic acid 0.4 mg as part of routine antenatal care follow-up throughout the pregnancy [11]. Studies have also witnessed that improving maternal hemoglobin level during pregnancy can reduce the risk of maternal mortality and morbidity from postpartum hemorrhage [4].

Cognizant of these, the Federal Government of Ethiopia established the national nutrition packages to improve micronutrient deficiencies among pregnant women by ensuring access to routine nutritional counseling and supplementations as dewarming, insecticide-treated nets, and iron-folic acid supplementations during perinatal care [12]. Even though considerable efforts have been made to reduce the prevalence of anemia in the country, the Ethiopia demographic health survey of 2016 indicated that 29% of pregnant women and 24% of female reproductive age groups were still anemic [13]. To attain the second global nutrition target set of a 50% decrease of anemia among reproductive age of women by the year 2025, it was agreed that a combination of key programs and strategies that are tailored to local conditions, taking into account the definite cause and frequency of anemia in a given area and group of population, is required [1, 6]. However, there was a paucity of information that elucidates the hemoglobin level of pregnant women in low-income countries like Ethiopia especially in the study area, rural communities. Therefore, this study is aimed at assessing the hemoglobin level and associated factors among pregnant women in rural communities of Jimma zone, Southwest Ethiopia.

## 2. Methods and Materials

### 2.1. Study Design, Setting, and Participants

A community-based cross-sectional study design was carried out in Mana and Seka Chekorsa districts of Jimma zone, Southwest Ethiopia from June 1 to 30, 2020. The zone was found 345 km away from the capital city Addis Ababa. Jimma zone has two known agroecological (coffee growing and food crop growing) districts [14, 15]. Accordingly, Mana was selected from mainly coffee growing districts and found at 1911 meters of/altitude above sea level with an estimated total population of 160,096, of whom 79,615 were women. The district has 26 kebeles (small administrative units) including Yebu and Bilida Towns. The other selected district was Seka Chekorsa from principal grain and food crop producing districts. It had an altitude between 1580 to 2560 meters above sea level and an estimated total population of 336,277, of whom 167,414 were females. This district has 37 kebeles including the two town kebeles of Seka. The study was conducted among first-trimester pregnant mothers 15-49 years of age.

### 2.2. Inclusion and Exclusion Criteria

All randomly selected first-trimester pregnant women who were living in the area at least for the last six months before the survey were included in the study. First-trimester pregnant women with known preexisting or current medical conditions were excluded.

### 2.3. Sample Size Determination and Sampling Technique

The sample size was calculated using G power 3.1 software considering the following assumptions: *t*-tests-correlation: point biserial model, two tail, effect size ∣*ρ* | = 0.15, *α* err prob 0.05, power (1 − *β* err prob) = 0.80, then, the total sample size was 343. The final sample size was 378 after adding a 10% nonresponse rate. A list of all kebeles of both districts (37 from Seka Chekorsa and 26 from Mana) was made, and a unique identification number was assigned to each. Then, this identification number was used as a sampling frame to select kebeles. From a total of 63 kebeles listed, 21 were selected by a simple random sampling method. After all, the sample size was proportionally allocated to each kebele. The sampling frame of the study participants was prepared after home to the home identification and recording of all eligible pregnant mothers. Then, first-trimester pregnant women were selected using systematic random sampling. Pregnant women who were absent from home were revisited on the next day. If a woman was still absent from her house during the interview, eligible pregnant women in the next house in the serial number were interviewed. The pregnancy was confirmed by ultrasound scanning and pregnancies that were diagnosed as nonviable or had incurable deformities were referred to the nearest health facility for management.

### 2.4. Data Collection Instruments, Techniques, and Measurements

The data were collected by a pretested, interviewer-administered structured questionnaire. It was collected by experienced and trained eight BSc holder midwives who could speak the local language “Afan Oromo.”

### 2.5. Measurements of Hemoglobin Level

The hemoglobin level of women was measured by using HemoCue AB Sweden 301(HemoCue AB, Angelhom, Sweden). The machine was a precalibrated instrument designed for the measurement of hemoglobin concentration. The blood sample was collected from each participant using a figure pricking method to collect three drops of blood from the left ring finger. The first and second drops of blood were wiped away. Then, the third drop was used for testing the hemoglobin level. The blood was drawn through microcuvettes and inserted into the HemoCue machine, and the results were recorded.

### 2.6. Mid-Upper Arm Circumference Measurement

Mid-upper arm circumference measurement was an average of three measurements to the adjacent cent-meter using a flexible nonelastic tape. It is midway between the tip of the shoulder (acromion process) and the tip of the elbow (olecranon process) of the left arm hanging freely.

### 2.7. Minimum Dietary Diversity–Women (MDD-W)

The data of minimum dietary diversity score were collected by using a 24-hour dietary recall method according to FAO's 2016 guideline [16]. To each food group that a woman ate in 24 hrs of the day before data collection score of “1” was given and otherwise “0.” The dietary diversity score was made by counting the number of scores of food groups. For one food group, only a score of “1” was given and sum up all without considering the number of foods eaten in the same food group. Finally, a woman who had got 5 scores or more out of ten was categorized as adequate and otherwise inadequate dietary diversity. The ten food groups used were starchy foods, pulses, nuts and seeds, milk and milk products, meat, eggs, dark green leafy vegetables, vitamin a-rich fruits and vegetables, other vegetables, and other fruits.

### 2.8. Data Quality Control

The questionnaire was first prepared in English and translated into the local language “*Afan Oromo*” by fluent speakers of both languages. Then, it was translated back to English by the third expert to ensure its consistency. To ensure the quality of data, three days of intensive training were given for data collectors and supervisors on the objective of the study, methods of data collection, anthropometric measurement, blood sample collection, and data recording methods. A practical test was administered for data collectors to make sure that the skill was appropriately transferred. In addition to this, two trained supervisors were assigned to give on-site support and oversee the completeness of the collected data overnight. A pretest was conducted on 5% of the total sample size in a nonselected setting that had similar characteristics of respondents in the Kersa district of Jimma Zone.

### 2.9. Data Processing and Analysis

Data were checked for consistency, accuracy, and completeness. Then, it was entered into Epi-data version 3.1 and exported to STATA version 13 for data cleaning and analysis. Data cleaning was made by checking outliers, ascending and descending order. Descriptive statistics such as central tendency measurements and variation: mean, frequency, percentages, and standard deviations were used to describe the study subjects. Major assumptions for binary and multivariable linear regression analyses were checked by examining the residuals for the linearity assumption using a scatter plot for all numerical predictor variables and the dependent variable (mean hemoglobin level). All variables were normally distributed and assessed visually using a histogram. Moreover, homoscedasticity was checked by using a scatter plot, and there was no heteroscedasticity or clear pattern on the plot. Likewise, multicollinearity was checked using a variance inflation factor, and there was no interaction. Simple linear regression analysis was performed for each independent variable against the dependent variable. Variables with *P* value < 0.25 were considered for multivariable linear regression analyses to control the possible effects of a confounder. Variables with a *P* value of 0.05 were declared significantly associated with the mean hemoglobin level of women in the final adjusted multivariable linear regression model. Unstandardized beta (*β*) coefficients along with 95% confidence interval (CI) were computed to assess the level of association and statistical significance in multivariable linear regression analysis. The wealth index was estimated as a composite indicator of living standards by considering 29 variables related to ownership of household durable assets (both productive and nonproductive assets). Assumptions of the principal component analysis were checked. The overall sampling adequacy (KMO > 0.5), Bartlett's test of sphericity (*P* ≤ 0.05), having commonality >0.5, not having the complex structure correlation ≥ 0.40 assumptions were checked and components that collectively explain more than 60% of the variance in the set of variables were used for generation of a continuous variable by summing up the principal components into one and tertile rank was made into rich, medium, and poor.

### 2.10. Ethical Consideration

Ethical approval was obtained from Jimma University Institutional Review Board (IRB) and Oromia Regional Health Bureau. A letter of permission was obtained from the Health office of Jimma Zone. After explaining the purpose and objective of the study, written informed consent and fingerprint (who were unable to read and write) were obtained from each participant. Confidentiality and privacy were kept throughout the study. Privacy of the respondents was maintained by interviewing them in an isolated room. The data were kept under a locked cabinet and were not disclosed to anyone except the investigators. The procedure constituted a minimal risk to the participants, and it was explained before the onset of the data collection process.

## 3. Results

A total of 367 pregnant women participated in the study with a response rate of 97%. The mean (SD±) age of the respondents was 23.77 (±4.80) years. A majority (96.73%) of the participants were Oromo by ethnicity and 90% Muslim religion followers. About half of them (48.5%) were attended some primary school grades whereas one quarter (21.8%) were unable to read and write. Most of the respondents (72.76%) were housewives followed by merchants (21%) and daily laborers (2.72%). Almost all of the participants (99.72%) were married. Most of the respondents (72%) were using protected spring water for drinking. Large proportions (70.30%) of the participants were from the medium wealth tertile. The overall magnitude of anemia (hemoglobin level < 11 g/dl) among pregnant women was found to be 85 [23.16%, (95% CI: 18.3%-27.5%)] (Table 1).

### 3.1. Obstetrics and Pregnancy-Related Characteristics

All of the respondents were in first-trimester pregnancy with the majority (93.73%) of 8-13 gestational weeks. More than half of the respondents (63.22%) had less than two years of interpregnancy interval. Moreover, the majority of them had no history of stillbirth (91.55%) and cesarean section (97.55%). Less than a quarter (19.35%) of the respondents had a history of abortion (Table 2).

### 3.2. Dietary Practices of Pregnant Women

More than half (52.86%) of the respondents had fruits/vegetables in their garden either to consume or sell it. Most of the respondents (74.39%) did not change their diet during pregnancy. Besides, 20% of them had avoided some types of foods during pregnancy because of food taboos. About 83.11% of the women did not receive iron folate supplementation during their first trimester of pregnancy. Moreover, most of the respondents consumed coffee during the current pregnancy (82.29%) (Table 3).

### 3.3. Factors Associated with Maternal Hemoglobin Status

Simple linear regression analysis was performed for all independent variables. However, only ten variables, MUAC(mid-upper arm circumference), maternal education of completing grade 8, interpregnancy interval, having a history of stillbirth, meal frequency, coffee consumption, iron folate supplementation, own fruits/vegetables, were linearly associated with a hemoglobin level of a woman at *P* value < 0.05 (Table 4). The final adjusted multivariable linear regression analyses revealed that maternal hemoglobin level was positively associated with meal frequency [*β* = 0.409, (95% CI: 0.123, 0.694), *P* = 0.005], duration of interpregnancy interval [*β* = 0.089, (95% CI: 0.024, 0.154), *P* = 0.007], mid-upper arm circumference measurements [*β* = 0.139, (95% CI: 0.071, 0.207), *P* ≤ 0.001], and having fruits/vegetables [*β* = 0.557, (95% CI: 0.317, 0.796), *P* ≤ 0.001]. Whereas it was negatively associated with women having a history of still birth [*β* = −0.634, (95% CI: -1.061, -0.208), *P* = 0.004] and consuming coffee during their current pregnancy [*β* = −1.000, (95% CI: -1.317, -0.683), *P* ≤ 0.001] (Table 5).

## 4. Discussions

This study revealed that the mean (± SD) hemoglobin level of pregnant women in rural Southwest Ethiopia was 12.66 (±1.40) g/dl with a range of 9-18 g/dl. The current result obtained in this study was agreed with the findings reported in the previous studies carried out in the West Arsi Zone of Ethiopia 12.05 (±1.5) g/dl [17], Bsidimo Hospital 11.4 (±2.3) g/dl [18], Rural community of Eastern Ethiopia 11(±1.7) g/dl [19], Southern Ethiopia 11.9 (±1.4) g/dl [20], Debremarkos Hospital 12.65 (±2.82) g/dl [21], and Northwestern zone of Tigray region 11.21 (±1.8) g/dl. However, it was higher than the reports from Accra, Ghana 10.9 (±1.3) g/dl [22], Karnataka, India 9.6 (±1.63) g/dl [23], and the Kolar Taluk district of India 10.5 g/dl [24]. This difference might be because of the geographical and sample size variations across the studies.

The prevalence of anemia (hemoglobin level < 11 g/dl) among pregnant women observed in this study was 23.16% [2]. This was agreed with the findings from the Oromia region (24.09%) [25], Southern Ethiopia (23.2%) [20], and China (23.5%) [26]. However, it was much lower than similar studies reported from Southwest Ethiopia (31.4%) [27], Rural communities of Eastern Ethiopia (43.9%) [19], different parts of Ethiopia [13, 18, 28–30], Democratic Republic of Congo (53.4%) [31], and India (62.3%) [24]. On the other hand, it was higher than the findings reported from Adama Hospital (14.9%) [32], St. Paul's Hospital (11.6%) [33], Addis Ababa (18.4%) [34], the Central Zone of Tigray region Ethiopia (16.88%) [35], Tanzania (18%) [36], and Turkey(7%) [37]. The possible explanation could be because of the study settings and the geographical variations. In addition to this, using different cut-off points and hemoglobin level measurements for anemia might also have resulted in variation of prevalence rate.

The current study also demonstrated that pregnant women who increased one meal frequency during pregnancy increased mean hemoglobin level by 0.41 g/dl. This finding was supported by the studies conducted in the rural communities of Eastern Ethiopia [19], the Northwestern Zone of Tigray [38], and the Central Zone of Tigray [35]. This could be explained by the fact that increasing meal frequency during pregnancy enhances the probability of consuming iron-rich diversified diets and decreases the risk of anemia [20].

A one-year increment of the interpregnancy interval increased the mean hemoglobin level of a woman by 0.09 g/dl in the current study. This observation was comparable with the findings of similar previous studies conducted in the Asossa zone of Ethiopia [39], Tigray region North Ethiopia [40], a systematic review and meta-analysis done in Ethiopia [30], and Tanzania [41]. This implied that a woman with a short interpregnancy interval was more likely to develop anemia during the current pregnancy than the others. This might be explained by the fact that close succession of pregnancies worsens the mother's nutritional status by depleting the total body iron of the women and become anemic, because of inadequate time to recover from the physiological stress of the proceeding pregnancy before becoming subject to the next [42].

This study also revealed that per one-centimeter increment of maternal mid-upper arm circumference measurement increases the mean hemoglobin level of a woman by 0.14 g/dl. The current finding was supported by the studies conducted in the urban area of Eastern Ethiopia [28], the Rural part of Jigjiga [29], pregnant women of Ethiopia [43], and Rural Oromia residents [25]. This might be because of the reason that undernourished pregnant women had a higher probability of being micronutrient deficient and anemic [44].

Likewise, being own fruits/vegetables in their garden was increasing maternal hemoglobin level by 0.5 g/dl in this study. Other similar studies from different parts of Ethiopia, West Arsi Zone [17], and Central Ethiopia [45], Assosa zone, and Northwest Ethiopia (27) were comparable reported findings. This result might be because of the reason that women who own fruits/vegetables might be easily accessible and consume vitamin C and A source fruits/vegetables which are enhancers of gastrointestinal iron absorption [46]. The soluble complex compound formed by the reaction of vitamin A and *β*-carotene with iron in the lumen of the intestine may avoid the inhibitory effect of polyphenols and phytates on the absorption of iron [47].

In this study, we also observed that there was a negative linear association between pregnant women's mean hemoglobin level and having a history of stillbirth. Thus, having a history of stillbirth was decreased the mean hemoglobin level of the women by 0.63 g/dl. Our findings agreed with other study reports from Addis Ababa [48], the Democratic Republic of Congo [31], India [49], and England [50]. This might be explained by maternal anemia impaired placental function by increasing fetoplacental vasculogenesis and angiogenesis as an adaptive response. Thus, it decreases the nutrients supplementation to the fetus and leads to intrauterine growth retardation and stillbirth [51, 52].

Coffee consumption was another variable negatively associated with the hemoglobin level of the mother. Accordingly, coffee consumption during the current pregnancy decreased the hemoglobin level of pregnant women by 1.00 g/dl. This present result supported the report of previous studies from Central Ethiopia [45], Kacha Bira district of Southern Ethiopia [53], Durame town Ethiopia [54], Debremarkos Hospital [21], and Kartum Sudan [55]. This might be because of the reason that coffee contains phenolic acid such as a chlorogenic acid that inhibits the absorption of nonhaem iron, which is necessary for red blood cell production [56, 57].

In this study, analyses of different sociodemographic, obstetrics, and dietary factors that might be associated with the hemoglobin level of the women were carried out. Contrary to the previous findings, this study did not observe a statistically significant association between maternal hemoglobin level and factors like maternal age [26, 27, 39, 58, 59], grand multiparty [17, 20, 27, 30, 60], iron folate supplementations [20, 29, 33, 58, 61], income/wealth index [20, 26, 40, 59, 60], maternal education level [59, 61], and Khat chewing [19]. The research team recommends further study to evaluate the relationship between the aforementioned variables.

This study is not merely done without limitation. The information gathered was based on the report of the mother that it was likely to have social desirability and recall biases. Some predictors of anemia like some micronutrient deficiency and the presence of parasitic and malarial infections were not measured due to feasibility issues.

## 5. Conclusions

Anemia was found to be a moderate public health problem in the study area. Meal frequency, interpregnancy interval, mid-upper arm circumference measurement, having fruits/vegetables, coffee consumption, and having a history of stillbirth were linearly associated with the hemoglobin level of pregnant women. Therefore, nutritional counseling should focus on the necessity of at least one extra meal, promotion of fruits/vegetable consumption, and improving the nutritional status of the women by care providers during antenatal care follow-up. Moreover, early screening and management of women with a history of stillbirth for anemia are also essential.

## Figures and Tables

**Table 1:** Sociodemographic and economic characteristics of pregnant women in rural Jimma Zone, Southwest Ethiopia, 2020, (*n* = 367).

Variable	Anemia (<11 g/l)		Total (%)	*X* ^2^	*P* value
	No (%) (*n* = 85)	Yes (%) (*n* = 282)			
Age in years					
15-20	90 (24.52)	36 (9.80)	126 (34.33)	6.22	0.18
21-24	48 (13.08)	12 (3.27)	60 (16.35)		
25-29	108 (29.43)	28 (7.63)	136 (37.06)		
30-34	20 (5.45)	8 (2.18)	28 (7.63)		
35-49	16 (4.36)	1 (0.27)	17 (4.63)		
Ethnicity					
Oromo	271 (73.84)	84 (22.89)	355 (96.73)	2.18	0.70
Amhara	4 (1.09)	1 (0.27)	5 (1.36)		
Dawuro	4 (1.09)	0 (0)	4 (1.09)		
Yem	3 (081)	0 (0)	3 (0.81)		
Religion					
Muslim	250 (68.12)	83 (22.62)	333 (90.74)	6.63	0.03
Orthodox	26 (7.08)	1 (0.28)	27 (7.36)		
Protestant	6 (1.64)	1 (0.27)	7 (1.91)		
Maternal education					
Unable to read and write	59 (16.07)	21 (5.73)	80 (21.80)	1.13	0.95
Elementary grades	136 (37.06)	42 (11.44)	178 (48.50)		
Completed grade 8	42 (11.44)	10 (2.73)	52 (14.17)		
High school grades	21 (5.72)	5 (1.36)	26 (7.08)		
Collage and above	24 (6.53)	7 (1.91)	31 (8.44)		
Maternal occupation					
Merchant	59 (16.07)	18 (4.13)	77 (20.20)	2.99	0.55
Housewife	203 (55.32)	64 (17.43)	267 (72.75)		
Governmental employee	5 (1.36)	2 (0.55)	7 (1.91)		
Student	6 (1.63)	0 (0)	6 (1.63)		
Daily laborer	9 (2.46)	1 (0.26)	10 (2.72)		
Marital status					
Married	281 (76.56)	85 (23.16)	366 (99.72)	0.30	0.58
Widowed	1 (0.28)	0 (0)	1 (0.28)		
Husband's occupation					
Merchant	76 (20.71)	21 (5.72)	97 (26.43)	10.63	0.10
Farmer	122 (33.25)	40 (10.89)	162 (44.14)		
Governmental employee	18 (4.9)	0 (0)	18 (4.9)		
Daily laborer	57 (15.53)	9 (2.45)	66 (17.98)		
Private employee	16 (4.36)	8 (2.18)	24 (6.54)		
Family size					
Less than five	236 (64.30)	71 (19.35)	307 (83.65)	0.001	0.97
Six and above	46 (12.53)	14 (3.82)	60 (16.35)		
Household head					
Man	271 (73.84)	83 (22.62)	354 (96.46)	0.45	0.49
Woman	11 (3.00)	2 (0.54)	13 (3.54)		
Drinking water source					
Public tape	35 (9.54)	14 (3.81)	49 (13.35)	0.05	0.56
Protected spring	206 (56.13)	58 (15.80)	264 (71.93)		
Pipe water	29 (7.90)	11 (3.00)	40 (10.90)		
River	12 (3.27)	2 (0.54)	14 (3.81)		
Household wealth index					
Rich	21 (5.72)	6 (1.64)	27 (7.36)	1.42	0.49
Medium	202 (55.04)	56 (15.26)	258 (70.30)		
Poor	59 (16.08)	23 (6.26)	82 (22.34)		

**Table 2:** Obstetrics and pregnancy-related characteristics of pregnant women in rural Jimma Zone, Southwest Ethiopia, 2020, (*n* = 367).

Variable	Anemia (<11 g/l)		Total (%)	*X* ^2^	*P* value
	Yes (*n* = 85)	No (*n* = 282)			
Gestational age					
<8weeks	5 (1.36)	11 (3.00)	16 (4.36)	0.61	0.43
8 to 13 weeks	80	271	351 (95.64)		
Inter pregnancy interval					
≤2 years	37	95	132 (35.96)	2.74	0.09
>2 years	48	187	235 (64.03)		
Parity of women					
Primi Para	26	79	105 (28.61)	4.85	0.30
1–2 children	43	136	179 (48.77)		
3–4 children	11	56	67 (18.26)		
5 and more children	5	11	16 (4.36)		
History of abortion					
Yes	12	59	71 (19.35)	1.93	0.16
No	73	223	296 (80.65)		
History of stillbirth					
Yes	9	22	31 (8.45)	0.61	0.43
No	76	260	336 (91.55)		
History of cesarean section					
Yes	1	8	9 (2.45)	0.75	0.38
No	84	274	358 (97.55)		
Has nausea or vomiting					
Yes	32	120	152 (41.42)	0.64	0.42
No	53	162	215 (58.58)		

**Table 3:** The dietary practices of pregnant women in rural Jimma Zone, Southwest Ethiopia, 2020, (*n* = 367).

Variable	Anemia (<11 g/dl)		Total (%)	*X* ^2^	*P* value
	Yes *n* = 85 (%)	No *n* = 282 (%)			
Own fruit/vegetable garden					
Yes	38 (10.35)	156 (42.51)	194 (52.86)	2.95	0.08
No	47 (12.81)	126 (34.33)	173 (47.14)		
Used fruit/vegetables					
Sell all	0 (0)	2 (1.02)	2 (1.02)	0.57	0.74
Sell some	23 (6.27)	82 (47.30)	105 (53.57)		
Consumed all	20 (5.45)	69 (39.96)	89 (45.41)		
Changed food habit during pregnancy					
Yes	26 (7.08)	68 (18.53)	94 (25.61)	1.43	0.23
No	59 (16.07)	214 (58.32)	273 (74.39)		
Avoid food during pregnancy					
Yes	19 (5.18)	53 (14.44)	72 (19.62)	0.52	0.46
No	66 (17.98)	229 (62.40)	295 (80.38)		
Types of foods avoided					
Sugar cane	9 (2.46)	23 (41.98)	32 (44.44)	0.08	0.96
Avocado	5 (1.36)	15 (25.02)	20 (26.39)		
Tomato	6 (1.63)	15 (29.54)	21 (29.17)		
Reason for food avoidance					
Big baby	16 (4.35)	39 (72.03)	55 (76.39)	2.78	0.42
Maternal obesity	0 (0)	2 (2.78)	2 (2.78)		
Discolor the fetus	0 (0)	5 (6.94)	5 (6.94)		
Difficulty during labor	3 (0.81)	7 (13.07)	10 (13.89)		
Iron folate supplementation					
Yes	9 (2.46)	53 (14.44)	62 (16.89)	3.13	0.07
No	76 (20.70)	229 (62.40)	305 (83.11)		
Coffee consumption					
Yes	71 (19.34)	231 (62.95)	302 (82.29)	0.11	0.73
No	14 (3.81)	51 (13.90)	65 (17.71)		
Khat chewing					
Yes	19 (5.18)	68 (18.53)	87 (23.71)	0.11	0.73
No	66 (17.98)	214 (58.31)	280 (76.29)		
Alcohol consumption					
Yes	0 (0)	3 (0.82)	3 (0.82)	0.91	0.34
No	85 (23.16)	279 (76.02)	364 (99.18)		

**Table 4:** Simple linear regression model predicting hemoglobin level among pregnant women in Jimma Zone, Southwest Ethiopia (*n* = 367).

Model	Unstandardized coefficients		*P* value	95% confidence interval	
	*β*	Std. error		Lower bound	Upper bound
Age (yrs)					
15-20	Ref				
21-24	0.07	0.21	0.74	-0.35	0.50
25-29	0.14	0.17	0.39	-.019	0.48
30-34	-0.32	0.29	0.26	-0.89	0.24
35-49	0.17	0.36	0.63	-0.53	0.88
Religion					
Muslim	0.61	0.53	0.25	-0.43	1.66
Orthodox Christian	0.52	0.59	0.37	-0.63	1.69
Protestant	Ref.				
Maternal education					
Cannot read and write	Ref				
Elementary school	0.01	0.18	0.99	-0.35	0.37
Complete grade 8	0.51	0.24	0.03^∗^	0.02	0.99
High school	0.20	0.31	0.50	-0.40	0.82
Collage and above	-0.05	0.29	0.84	-0.63	0.52
Gravida	0.02	0.04	0.54	-0.06	0.11
Nausea and vomiting					
Yes	0.17	0.14	0.23	-0.11	0.46
No	Ref				
Food taboo					
Yes	0.22	0.18	0.22	-0.13	0.58
No	Ref				
Iron folate received					
Yes	1.28	0.18	<0.001^∗^	0.92	1.64
No	Ref				
Meal frequency	0.54	0.17	0.002^∗^	0.20	0.88
Interpregnancy interval	0.14	0.03	<0.001^∗^	0.07	0.02
History of stillbirth					
Yes	-0.86	0.25	0.001^∗^	-1.37	-0.36
No	Ref.				
MUAC	0.18	0.04	<0.001^∗^	0.10	0.26
Consumed coffee					
Yes	-1.22	0.17	<0.001^∗^	-1.57	-0.86
No	Ref.				
Alcohol consumed					
Yes	0.20	0.80	0.80	-1.38	0.88
No	Ref.				
Khat chewing					
Yes	0.03	0.17	0.85	-0.30	0.36
No	Ref.				
Own fruits/vegetables					
Yes	0.60	0.14	<0.001^∗^	0.32	0.88
No	Ref.				

^∗^Significant at *P* < 0.05.

**Table 5:** Multivariable linear regression model predicting hemoglobin level among pregnant women in Jimma Zone, Southwest Ethiopia (*n* = 367).

Model	Unstandardized coefficients		*P* value	95% confidence interval		Multicollinearity test
	*β*	Std. error		Lower bound	Upper bound	^∗^VIF
(Constant)	9.89	0.95	<0.001	8.02	11.76	
Meal frequency	0.40	0.14	0.005	0.12	0.69	1.02
Interpregnancy interval	0.08	0.03	0.007	0.02	0.15	1.03
History of still birth	-0.63	0.21	0.004	-1.06	-0.20	1.01
MUAC	0.13	0.03	<0.001	0.07	0.20	1.03
Consumed coffee	-1.00	0.16	<0.001	-1.31	-0.68	1.05
Own fruits/vegetables	0.55	0.12	<0.001	0.31	0.79	1.02
Iron-folate supplement	0.96	0.16	<0.001	0.63	1.28	1.06

*β*: beta coefficient; MUAC: mid-upper arm circumference; *R*^2^: 0.332.

## Data Availability

The data used to support the findings of this study are available from the corresponding author on reasonable request.
